# Intracranial Myxoid Variant of Angiomatoid Fibrous Histiocytoma: A Case Report and Literature Review

**DOI:** 10.7759/cureus.4261

**Published:** 2019-03-18

**Authors:** Nooshin Ghanbari, Alexander Lam, Victor Wycoco, Gabriel Lee

**Affiliations:** 1 Surgery, Royal Perth Hospital, Perth, AUS; 2 Neurosurgery, Royal Perth Hospital, Perth, AUS; 3 Radiology, Royal Perth Hospital, Perth, AUS; 4 Neurosurgery, Sir Charles Gairdner Hospital, Perth, AUS

**Keywords:** intracranial afh, myxoid variant

## Abstract

Angiomatoid fibrous histiocytoma (AFH) is a rare and slow-growing soft tissue lesion that typically arises in the extremities of young patients.

Microscopically, AFH is characterized by pseudovascular, blood-filled spaces that are surrounded by a multinodular proliferation of spindle and/or round cells and lymphoid cuffs. However, there is a wide morphological spectrum, including a myxoid variant. Examples with a prominent myxoid matrix are rare and may pose great diagnostic difficulty.

Specific gene fusions have been found to play a significant role in AFH tumorigenesis. Gene fusions of Ewing sarcoma breakpoint region 1 (EWSR1) with members of the cAMP response element-binding protein family (CREB) of transcription factors (CREB1, activating transcription factor 1 (ATF1), and cAMP response element modulator (CREM)) have been described in histopathologically diverse mesenchymal neoplasms such as AFH, hyalinising clear cell carcinomas of salivary glands, primary pulmonary myxoid sarcoma, and clear cell sarcoma. Classically, EWSR1-CREB is known to be the prominent gene fusion in AFH. Recently, a small series of intracranial mesenchymal tumors with EWSR1-CREB family gene fusions has been reported. These tumors seem to show histologic, immunophenotypic, and cytogenic features similar to those observed in the myxoid variant of AFH; therefore, there is a debate on whether these tumors merely represent a variant of AFH or a novel entity.

This case report is of a 58-year-old woman presenting with the first episode of generalized seizure due to an extra-axial lesion with homogenous contrast enhancement in the right parietal lobe, which was initially diagnosed as a World Health Organization (WHO) grade I meningioma. Following a series of pathological investigations, the diagnosis of an intracranial myxoid variant of AFH was made. This case report illustrates the need to consider the myxoid variant of intracranial AFH in the differential diagnosis of meningioma-like tumors. A high index of suspicion is required if the meningioma behaves abnormally with a much higher recurrence rate.

## Introduction

Angiomatoid fibrous histiocytoma (AFH) is generally considered a slow-growing, rarely metastasizing mesenchymal tumor of uncertain differentiation, mostly arising in the extremities of children and young adults in the second or third decades of life [1]. Clinically, patients with AFH can experience systemic symptoms such as fever, anemia, and weight loss [2].

Histologically, typical AFHs are characterized by the multinodular proliferation of oval histiocytoid to spindle cells with syncytial growth, forming sheets, and vague bundles or whorls, accompanied by a dense, fibrous, pseudocapsule, pericapsular lymphoplasmacytic cuffing in varying proportions and central pseudoangiomatous spaces. The diagnosis of AFH is straightforward when these classic histological features are present. However, AFH may rarely display unusual clinicopathological features, including older age at presentation, occurrence outside somatic soft tissues, and variation in the structural patterns, stromal matrix, and cytomorphology [3]. Examples with a prominent myxoid matrix are also rare and may pose great diagnostic difficulty [4]. Given its histologic similarity to a variety of other neoplasms, it is likely that it has been misdiagnosed and subsumed under a variety of other neoplastic categories, such as a myofibroblastic tumor, poorly differentiated carcinoma, or meningioma [5].

Molecular genetic studies have revealed that AFH often demonstrates Ewing sarcoma breakpoint region 1-cAMP response element binding 1 (EWSR1-CREB1) fusion as a result of t(2:22) (q33;q12) and, less commonly, Ewing sarcoma breakpoint region 1-activating transcription factor 1 (EWSR1-ATF1) or fused in sarcoma-activating transcription factor 1 (FUS-ATF1) resulting respectively from t(12:22) (q13;q12) or t(12;16) (q13;p11) [3]. However, gene fusions of EWSR1 with the CREB family of genes (CREB1, ATF1, and cAMP response element modulator (CREM)) have been reported in various mesenchymal tumors, occurring in a variety of sites with extensive biological behavior. EWSR1-ATF1 fusions have been found in clear cell sarcoma and the hyalinising clear cell carcinoma of the salivary gland, whereas EWSR1-CREB1 fusion is the only fusion reported in primary pulmonary myxoid sarcoma to date [6]. Therefore, certain gene fusions are not adequate to confirm a diagnosis since these EWSR1-CREB fused lesions yield a large spectrum of neoplasms with different immunoprofiles and morphologies.

AFH was first described by Enzinger in 1979 as one of the five subtypes of malignant fibrous histiocytoma (MFH). The term "AFH" has been used rather than "angiomatoid MFH" because the prognosis is favorable [7]. Rare examples of intracranial classic AFH, extra-axial, and intra-axial parieto-occipital have been reported in the literature; however, none of these cases had myxoid features on histology [4].

In this report, we present a rare case of intracranial AFH with prominent myxoid features, initially diagnosed as meningioma, in a 58-year-old woman.

## Case presentation

**Figure 1:**
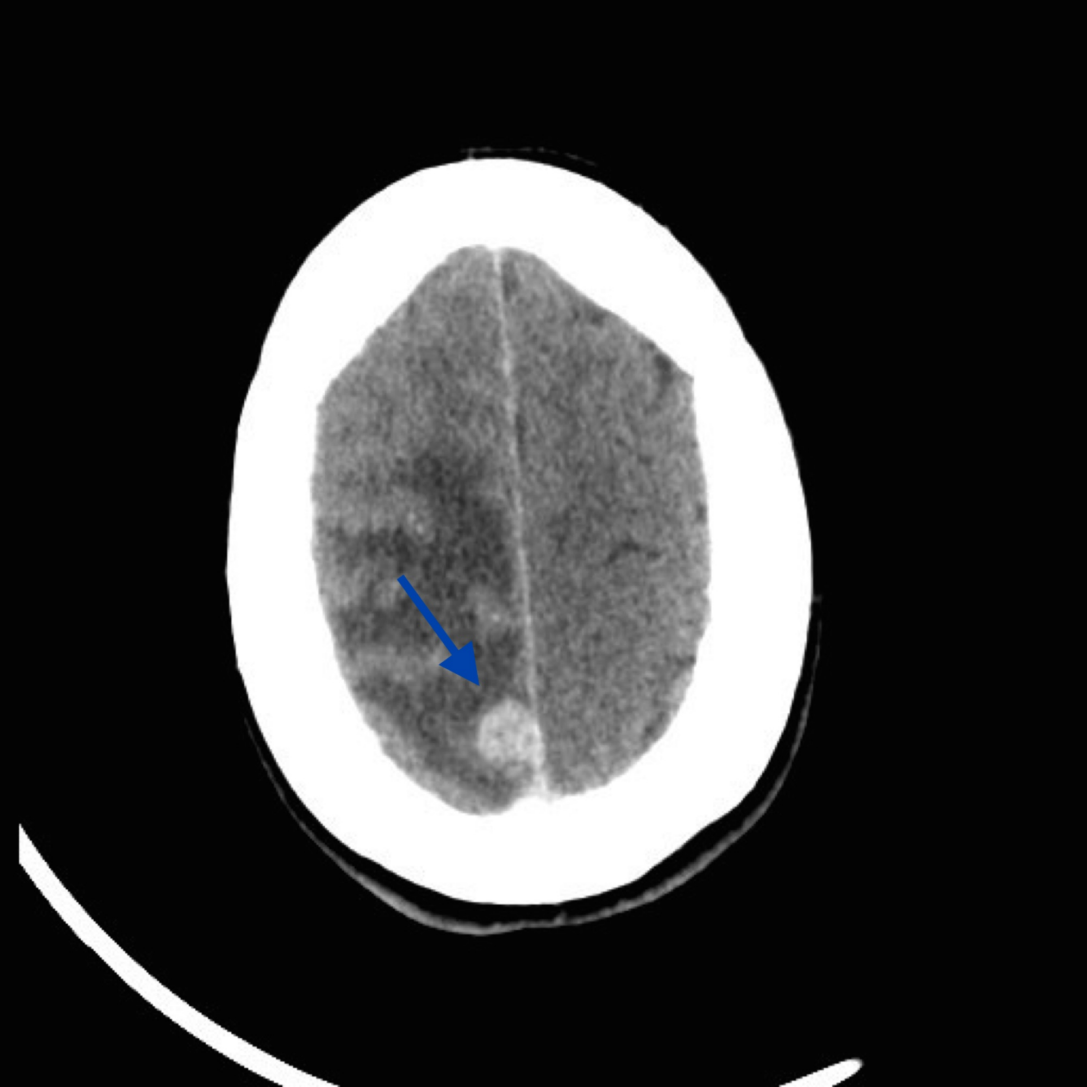
Preoperative axial CT scan with contrast: homogeneously enhancing parafalcine lesion in the right parietal region with surrounding vasogenic edema CT: computed tomography

**Figure 2:**
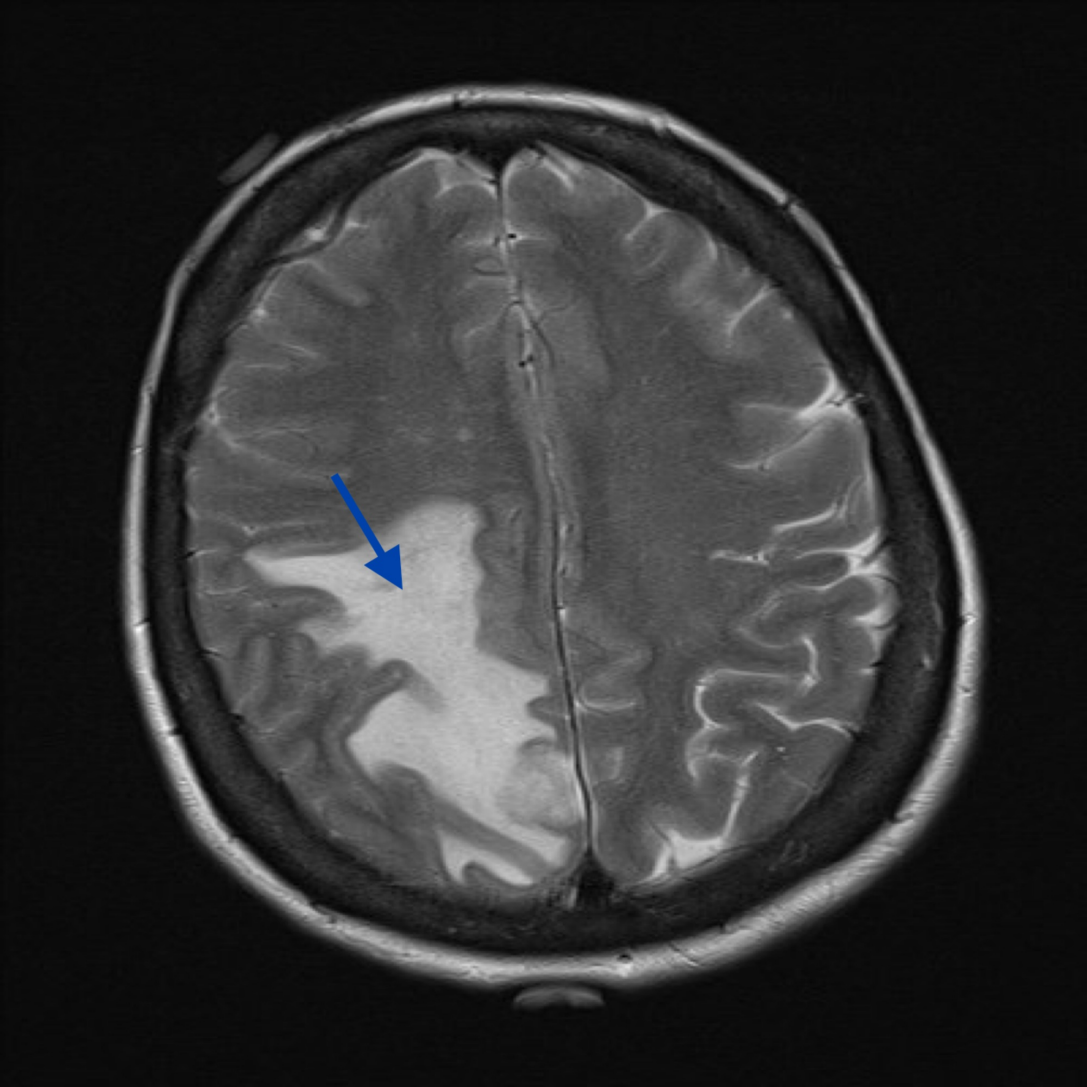
MRI, axial T2 demonstrating vasogenic edema associated with lesion MRI: magnetic resonance imaging

**Figure 3:**
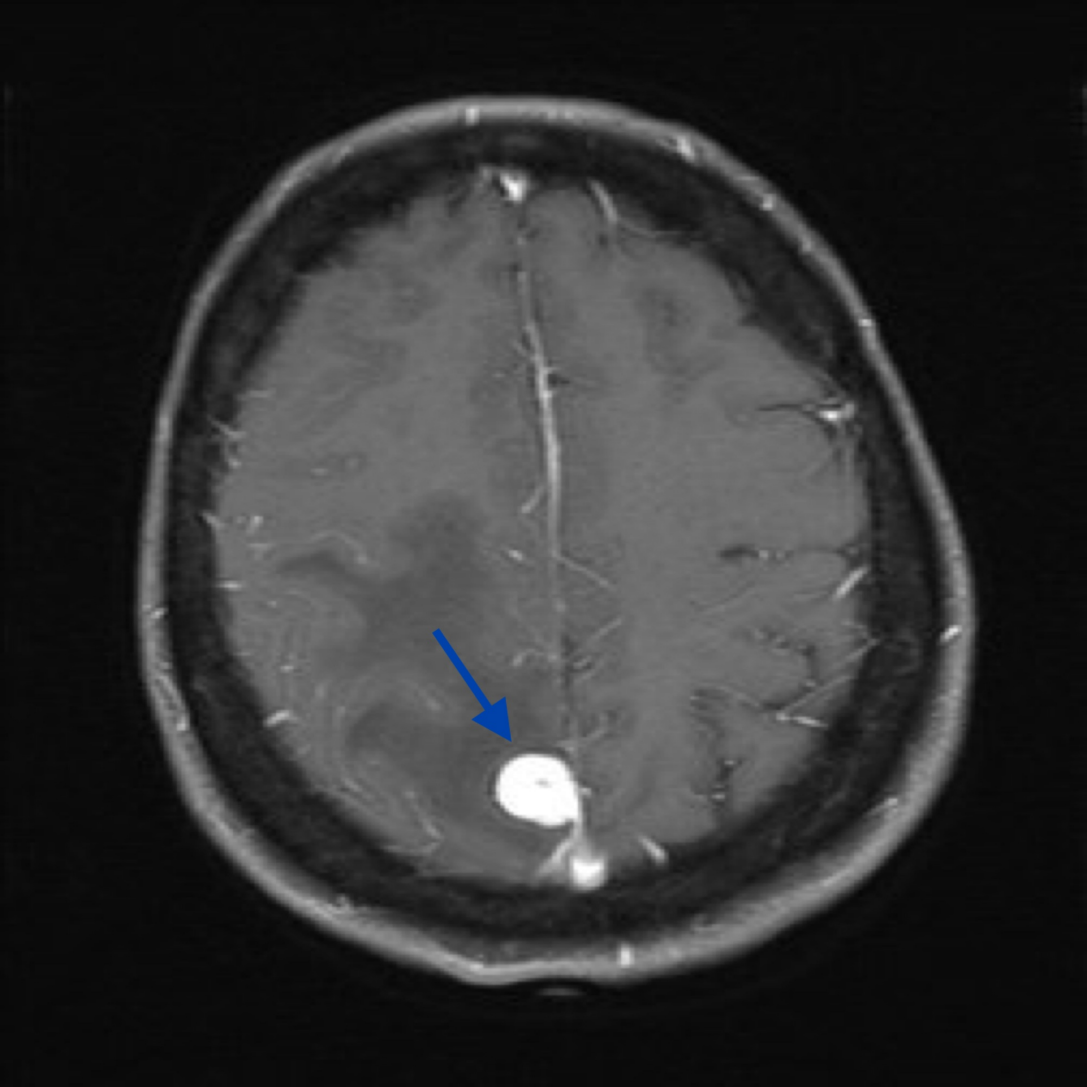
MRI, axial T1 post-gadolinium showing a well-demarcated extra-axial lesion with homogenous contrast enhancement and a small posterior dural tail MRI: magnetic resonance imaging

A 58-year-old woman presented with the first episode of a generalized tonic-clonic seizure. On examination, she had findings of mild left pyramidal hemiparesis associated with upper motor neuron signs. Brain resonance imaging (MRI) studies revealed a 16-mm right parafalcine,extra-axial lesion with homogenous contrast enhancement. There was surrounding vasogenic edema in the adjacent parietal and posterior frontal white matter (Figures 1-2). The lesion was thought to be extra-axial with a narrow dural base (Figure 3).

Initial body computed tomography (CT) revealed pretracheal and precarinal lymphadenopathy, although there was no definite primary lesion elsewhere. There was a remote history of a benign breast lesion. Provisional diagnosis favored a meningioma, although a metastasis remained in the differential, given the volume of vasogenic edema and mediastinal lymphadenopathy.

The patient underwent a parasagittal craniotomy and Simpson grade-two excision of the tumor. A small component of residual tumor was left deliberately, as it was adherent to the wall of the superior sagittal sinus. She recovered well following surgery, without any neurological deficits.

A histological examination revealed that the neoplastic cells had moderately pleomorphic nuclei, predominantly spindle in outline, with occasional plump and polygonal forms and eosinophilic cytoplasm. The cells were arranged in a fascicular pattern by variable amounts of myxoid stroma with a low mitotic rate (4/10 high power fields (HPFs)) but necrosis was absent. A well-developed and prominent peripheral lymphoid cuff was present. Immunohistochemically, the tumor cells were positive for desmin, anaplastic lymphoma kinase 1 (ALK1), vimentin, and epithelial membrane antigen (EMA).

Based on these findings, no final diagnosis was suggested. The differential diagnosis was reported between an atypical meningioma and an inflammatory myofibroblastic tumor (IMT). In view of the difficulties resolving the differential diagnosis, the case was referred for international consultation. The outcome was that the lesion had meningioma characteristics.

Due to the abnormal progression of the tumor and uncertainty about the diagnosis, the case was revisited and a fluorescence in situ hybridization (FISH) analysis was performed. The analysis confirmed the disruption of the EWSR1 gene at 22q12 and the disruption of the CREB1 gene at 2q33, without disruption of the ATF gene at 12q13. Based on these cytogenetic findings, in conjunction with histomorphology and immunophenotype, a final diagnosis of a myxoid variant of AFH was confirmed.

**Figure 4:**
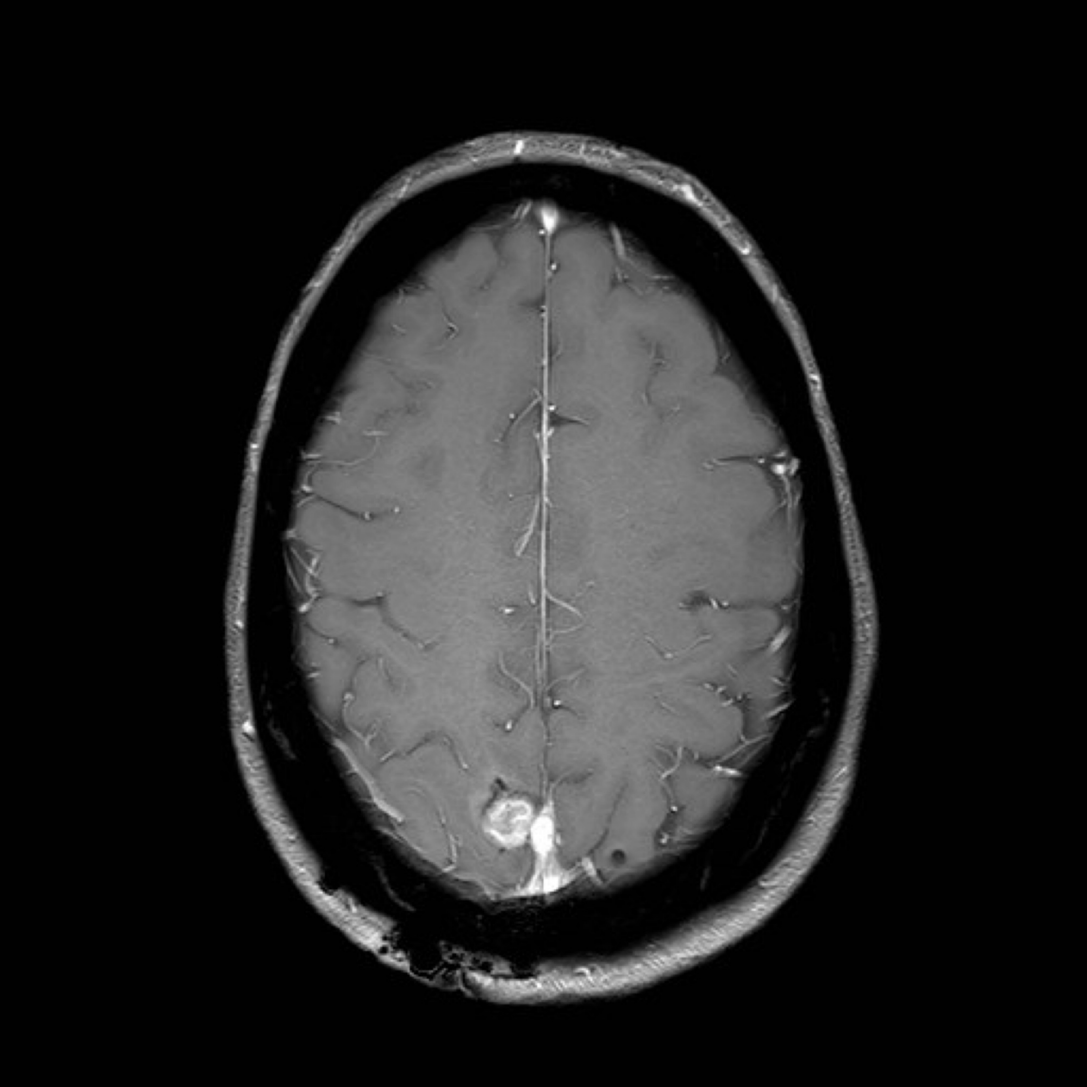
Axial T1 MRI FFE radiograph three months post-operation, showing a small nodular enhancement in the surgical cavity consistent with intraoperative observation of residual tumor MRI: magnetic resonance imaging; FFE: fast field echo

A follow-up MRI scan at three months revealed a small area of enhancement along the superior sagittal sinus, consistent with the intraoperative observation of residual tumor (Figure 4). The prior vasogenic edema had resolved, with no evident area of cortical thickening and white matter signal abnormality in the right middle frontal gyrus. Clinically, the patient remained asymptomatic since surgery and she reported no further seizure episodes. Subsequent positron emission tomography (PET) scan did not show active distant metastatic disease or fluoro-D-glucose (FDG) avidity in the mediastinal lymph nodes.

**Figure 5:**
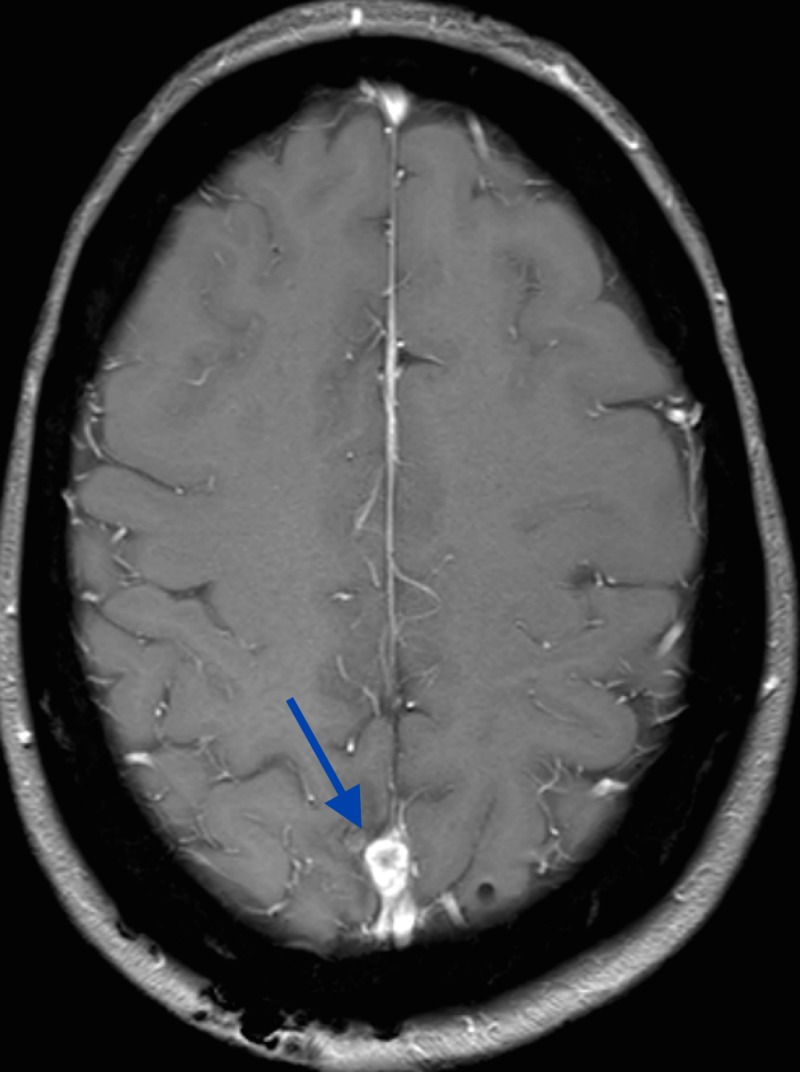
Axial T1 MRI demonstrates a recurrent right posterior falcine lesion measuring 11x7.5 mm MRI: magnetic resonance imaging

**Figure 6:**
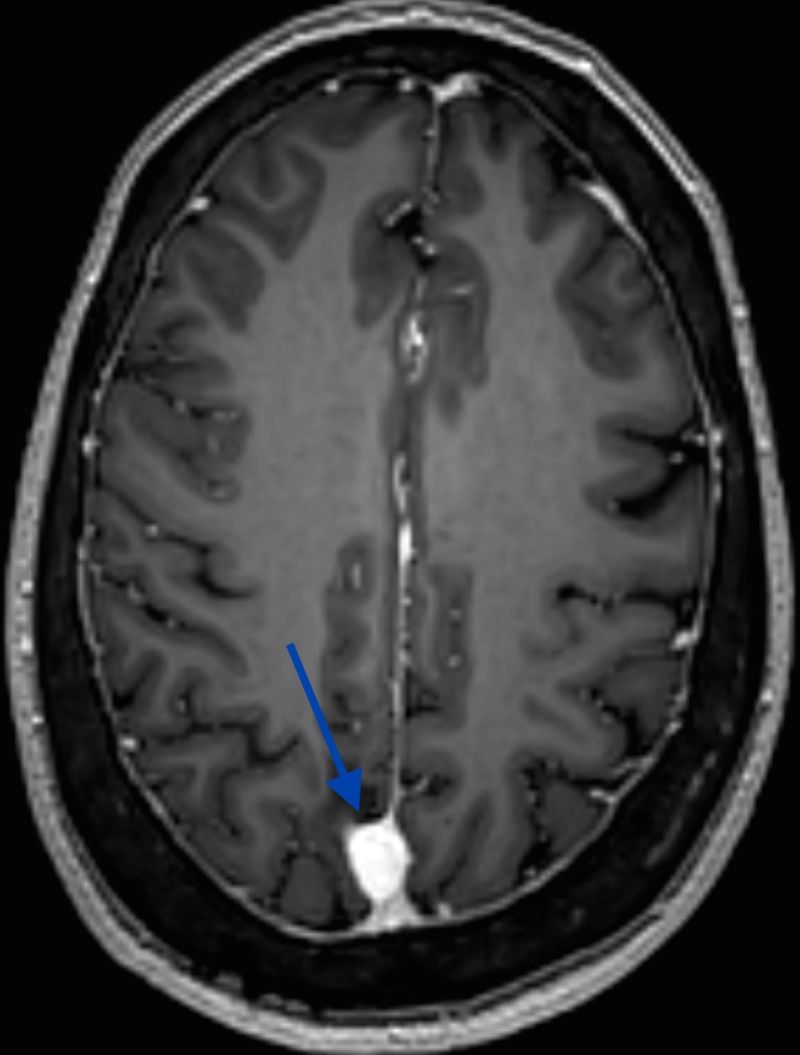
Axial T1-weighted MRI reveals the further progression of the right posterior parafalcine tumor (11x14 mm) MRI: magnetic resonance imaging

Findings on repeat CT scan of the whole body remained stable, including the pretracheal lymphadenopathy. No new lesions were identified on a follow-up PET scan. The recurrent intracranial lesion remained relatively stable on serial MRIs. The option of radiosurgery was discussed with the patient. She had elected to undergo radiosurgery treatment if subsequent MRI scans had demonstrated further progression (Figures 5-6).

## Discussion

AFH is a rare tumor primarily occurring in the extremities of children and young adults. It accounts for nearly 0.3% of all soft tissue tumors [8]. Most patients present in the first three decades of life, but there is a wide distribution of age. The myxoid variant of AFH carries low-to-intermediate malignant potential, with only rare patients developing local recurrence; no distant metastases have been reported. The majority of the EWSR1 rearrangements in the myxoid variant involve EWSR1-CREB1 with, less commonly, EWSR1-ATF1 resulting from t(12;22) (q13;q12). Histologically, it is characterized by a rich vascular network and vascular dilatation, epithelial cells, and a prominent myxoid background. A pseudocapsule and lymphoid aggregates are usually present [4].

Interestingly, a small series of intracranial myxoid mesenchymal tumors that harbor EWSR1-CREB family gene fusions have been reported recently. These tumors seem to show histologic, immunophenotypic, and cytogenic similarities to the myxoid variant of AFH but lack the typical capsular lymphocytic infiltrate and the prominent vascular component of the AFH myxoid variant. Therefore, there is a controversy related to whether these tumors merely represent a novel entity or simply an intracranial localization of the myxoid variant of AFH [4].

As mentioned previously, rare examples of intracranial classic AFH, extra-axial, and intra-axial parieto-occipital have been reported in the literature, however, none of these cases had myxoid features on histology.

The first confirmed primary intracranial AFH was reported by Dunham et al. in a 25-year-old healthy man who presented with headaches, nausea, vomiting, and visual disturbances [9]. Imaging demonstrated a large, cystic, heterogeneously enhancing, left occipital mass. Gross total resection was performed, with no further recurrence. The diagnosis of AFH was confirmed by positive genetic analysis via reverse transcription polymerase chain reaction (RT-PCR) for t(12;22) (q13;q12), resulting in clear cell sarcoma-like type 1 EWS/ATF-1 gene fusion, a genetic marker that has been linked to AFH [9].

In 2010, Ochalski et al. described a case of recurrent AFH with hemorrhagic features [1]. The patient was a 35-year-old male with right facial weakness, found to have an acute, left temporal intraparenchymal hemorrhage. The initial MRI scan showed a single 0.5x0.5 cm lesion, arising in the left mesial temporal area, with evidence of hemosiderin surrounding the tumor capsule. Angiography revealed no evidence of a vascular lesion. The patient underwent eight craniotomies and two radiosurgical ablations, however, he died of secondary hydrocephalus and intracerebral hemorrhage. Confirmatory diagnosis of AFH was established when the resection specimen showed characteristic histopathological findings of a fibrous pseudocapsule [1].

Hansen et al. reported an intracranial AFH in a 17-year-old patient with migraine headaches, blurry vision, and anemia [10]. Brain MRI showed a bilobed extra-axial parieto-occipital lesion. The tumor was initially misdiagnosed as rhabdoid meningioma, but the lesion lacked somatostatin receptor 2A (SSTR2A), which is usually expressed in meningioma. Interestingly, FISH analysis for EWS- or FUS-rearrangements were negative. The RT-PCR analysis also did not reveal EWS/ATF1 translocation. Ultimately, external consultation confirmed a diagnosis of AFH on the basis of immunohistochemical and morphologic features [10].

Alshareef et al. published a case of intracranial AFH in a patient relatively old for the onset of diagnosis [8]. The patient was a 58-year-old female who presented with right facial weakness, pain, and numbness. MRI displayed a large, 6.1x4.8x2.9 cm, heterogeneous, extra-axial mass centered in the right porous trigeminus. Although initially presumed to be meningioma or trigeminal schwannoma, a molecular, immunohistochemical, and histologic evaluation resulted in the final diagnosis of AFH. The key features of the lesion included a thick fibrous pseudocapsule, reactivity for CD163 and CD138 antibodies, and EWSR1 gene rearrangement [8].

The most recent case of intracranial AFH was reported by Spatz et al. in a 22-year-old female with a first-time generalized seizure, visual field deficit, and headache [7]. Imaging showed a 3.1x3.1x2.6 cm, heterogeneous, extra-axial lesion in the right occipital region. The tumor was initially diagnosed as an aggressive meningioma, but histopathological studies revealed the co-expression of epithelial membrane antigen (EMA) and desmin and the presence of a lymphoid cuff, based on which a diagnosis of AFH was made.

Our current case of a 58- year-old female represents the myxoid variant of intracranial AFH with EWSR1-CREB1 fusion. Although initially diagnosed as a WHO grade I meningioma, further consultation, including a FISH analysis, ultimately resulted in the final diagnosis of a myxoid variant of intracranial AFH. Key features included a prominent peripheral lymphoid cuff in a myxoid stroma and EWSR1-CREB1 fusion. Expression of EMA and desmin, typically expressed in AFH, was positive in this case. Based on histopathology and immunohistochemistry, the principal differential diagnosis was reported between an atypical meningioma and an inflammatory myofibroblastic tumor (IMT). Although the cytomorphology and EMA staining pattern were in favor of meningioma, the presence of ALK positivity and the extent of desmin staining were unusual for meningioma. Furthermore, approximately 50% of the IMTs harbor a clonal translocation that activates the anaplastic lymphoma kinase (ALK)-receptor tyrosine kinase gene located at 2p23 [11]. However, the FISH analysis in our case did not show a significant disruption of the ALK gene at 2p23. Also, our tumor demonstrated significant EMA expression, which is unusual in IMTs outside of the genitourinary tract.

To date, little is known about the radiographic characteristics of intracranial AFH. This lesion appears to mimic more common extra-axial tumors on imaging and has been mistaken for meningioma and schwannoma [4]. As a result, it is necessary to consider that AFH can mimic meningioma or other tumors on images. Radiographic findings of our case initially pointed toward meningioma. Similar to meningioma, this AFH displayed as an extra-axial lesion with homogenous contrast enhancement and the presence of a small dural tail in T1 fluid attenuated inversion recovery (FLAIR) MRI. However, a metastasis remained in the differential, given the volume of vasogenic edema and mediastinal lymphadenopathy in the whole body CT scan.

Assessing the prognosis of intracranial AFH is a challenge due to its infrequency. The overall behavior of AFH in other organ systems is indolent, with local recurrence in up to 15% of cases. Less than 5% can cause metastatic disease, sometimes after multiple recurrences, and this is mainly to regional lymph nodes. Local recurrence is related to infiltrative margins and locations in the head and neck, probably due to the difficulty in obtaining adequate surgical clearance at this site [12]. No other factors have been established to predict metastasis or the prognosis except a wide excision resection of the lesion. Of the case reports published on intracranial AFH, one of the five reported cases showed significant recurrence [8]. Therefore, post-excisional monitoring is warranted. We suggest follow-up MRI scans at three-month intervals for one year and six-month intervals for the subsequent two years. This report also illustrates the importance of considering intracranial myxoid AFH in the differential diagnosis of meningioma-like tumors. Hence, a high index of suspicion is required if a meningioma behaves abnormally, with a much higher recurrence rate.

## Conclusions

An angiomatoid fibrous histiocytoma is an extremely rare intracranial tumor. To the best of our knowledge, mixed AFH has not been reported in the central nervous system. We believe there are more cases of intracranial myxoid AFH that are misdiagnosed due to its intrinsic morphological variability. A combined approach using molecular studies and immunohistochemistry is suggested to differentiate this tumor from its mimics. In addition, this rare tumor can mimic more common extra-axial tumors on imaging and it should be considered in the differential diagnosis of meningioma-like tumors. Furthermore, limited evidence makes absolute therapeutic recommendations difficult. Follow-up MRI scans at three-month intervals for one year and six-month intervals for the subsequent two years are recommended.
